# Familial risk for depression is associated with reduced physical activity in young adults: evidence from a wrist-worn actigraphy study

**DOI:** 10.1038/s41398-024-02925-9

**Published:** 2024-05-28

**Authors:** Carola Dell’Acqua, Simone Messerotti Benvenuti, Nicola Cellini, C. J. Brush, Alessandra Ruggerone, Daniela Palomba

**Affiliations:** 1 Department of General Psychology, University of Padua, Padua, Italy; 2 Padova Neuroscience Center (PNC), University of Padua, Padua, Italy; 3 Hospital Psychology Unit, Padua University Hospital, Padua, Italy; 4 Department of Movement Sciences, University of Idaho, Moscow, ID USA

**Keywords:** Human behaviour, Psychiatric disorders

## Abstract

Depression is characterized by reduced physical activity and sleep-wake cycle disturbances, often considered important features of the disease. While a few studies have suggested that self-reported reduced physical activity and sleep-wake cycle disturbances might both be linked to depression vulnerability, actigraphy-based measures in vulnerable samples remain largely unexplored. This study relied on actigraphy-based parameters to test whether these disturbances characterize depression vulnerability. Seven-day actigraphy data were collected from 20 (13 female) university students with a high vulnerability to depression, which was determined by the presence of a family history of the condition but no current symptoms, and 32 (21 female) controls with neither a family history of depression nor current depressive symptoms. Daily physical activity, namely gross motor activity, was quantified as average daily acceleration and time spent engaging in moderate-vigorous physical activity (MVPA). The sleep-wake cycle and circadian rhythms were assessed as total sleep duration per night (in hours), sleep within sleep period time (in hours), sleep efficiency (%), and relative amplitude (i.e., the difference between the activity during the day and the night, which reflects circadian rhythms amplitude). Results showed that individuals with a familial risk for depression exhibited reduced daily acceleration and time spent in MVPA relative to the control group, particularly on the weekend during their free time away from scheduled activities. On the other hand, the two groups were comparable in terms of sleep estimates. Taken together, reduced physical activity, but not sleep-wake disturbances, seem to be associated with vulnerability to depression and might be a viable target for identification and prevention efforts.

## Introduction

Depression is a mood disorder that affects psychological and physiological functioning causing significant functional impairment and represents a primary contributor to the global burden of disease [1, 2]. Hence, improving its early identification and developing strategies to prevent its onset has been highlighted as a core priority. To this aim, determining factors that characterize individuals with a greater risk of developing depression, but no current symptoms, is becoming of increasing interest [3, 4]. One well-established risk condition is having a first-degree relative with a history of depression, whereby individuals with a first-degree relative with a history of depression are three to five times more likely to develop depression themselves [5, 6]. Although the familial risk is well-established, it is still necessary to identify the mechanisms underlying this vulnerability.

Given the heterogeneity that encompasses depression, a way to better identify the vulnerability mechanisms linked to depression is to move beyond the categorical approach (i.e., considering heterogeneous clusters of symptoms) and, instead, adopt a dimensional approach [4, 7]. In this context, the Research Domain Criteria (RDoC) project, launched in the United States by the National Institute of Mental Health (NIMH), encourages the exploration of symptom dimensions to advance our understanding of vulnerability to psychopathology across multiple levels of analysis [3, 7, 8]. Among such dimensions, the Arousal/Regulatory Systems sustain the appropriate activation levels for energy balance and sleep-wake regulation [9]. Reduced physical activity has been documented in individuals with depression, aligning with some clinical features, risk factors, and negative health outcomes associated with the condition (e.g., loss of pleasure, apathy, obesity, diabetes, and cardiovascular diseases [10–14]). Depression is also characterized by psychomotor retardation and less daytime physical activity, aligning with the Sensorimotor Systems, a domain recently added to the RDoC matrix [14]. Overall, less physical activity possibly relates to the lack of energy, greater apathy, and less motivation to engage in activities that characterize those with depression [15–17]. A recent meta-analysis of 49 studies observed that participants who reported high levels of physical activity were 17% less likely to have depression compared to those who had low levels of physical activity [17]. In addition, physical activity is a protective factor against the emergence or worsening of depression [18–22]. Despite the importance of motor activity in depression and its risk, research has long been confined to self-reported measures, leading to a biased estimation of the motor disturbances in this population [23]. Consistent with the RDoC recommendation to explore multiple levels of analyses, recent studies have explored motor abnormalities in depression with ecological tools [24, 25]. Wrist-worn actigraphy, which is commonly used to assess the sleep-wake cycle, has been recently employed to assess motor activity, showing that depression is linked to reduced daily acceleration and time spent doing moderate-to-vigorous physical activity (MVPA) [25–31]. Moreover, reduced physical activity in clinical samples was exclusively captured by actigraphy-based measures, but not self-reported questionnaires [27]. Nonetheless, to date only a few studies have relied on actigraphy to investigate motor disturbances as vulnerability indicators of depression in at-risk samples with no current depressive symptoms [25, 32, 33]. The lack of rigorous measures and systematic research on physical activity disturbances in at-risk samples does not allow for determining whether these disturbances might represent an underlying mechanism related to depression risk [23].

The Arousal/Regulatory Systems of the RDoC are also responsible for sleep-wake regulation. Depression has long been associated with sleep-wake disturbances, whereby those with depression show irregular patterns of sleep onset and offset associated, among others, with fatigue, somatic symptoms, and metabolic changes [13]. Indeed, given that behavioral and physiological processes are tied together within a 24 h internal biological clock, it is not surprising that depression is not only associated with physical inactivity but also with abnormal sleep-wake patterns [26, 27, 30, 34–37]. Circadian rhythm alterations and sleep-wake disturbances can be ascribed to the Arousal and Regulatory Systems (ARS) of the RDoC [38]. Particularly, those with depression tend to show altered circadian rhythms, such that they typically belong to a self-reported eveningness chronotype (i.e., favor waking up later and staying up late) [39]. Circadian rhythms regulate physical and behavioral changes over the 24 h cycle and are typically altered in terms of timing [40, 41]. This results in sleep deficits, such as delayed sleep onset, reduced sleep time, and enhanced wake-after-sleep onset in depression [26]. Particularly, individuals with depression are characterized by reduced sleep efficiency (i.e., total sleep duration divided by the time difference between sleep onset and wake-up time) and reduced relative amplitude (i.e., the amplitude between the activity during the day and the night, whereby lower relative amplitude relates to a dampened circadian rhythms amplitude and suggests lower activity during the day and greater activity during the night) [26, 42, 43]. To explain the interrelation between sleep-wake disturbances and depression, several neurobiological explanations have been put forward, including abnormalities in arousal [44], endocrine and monoamine functioning [44]. Moreover, twin studies have proposed that genetic variations might influence circadian rhythms, suggesting a partial heritability of sleep-wake irregularities [45]. Sleep-wake cycle disturbances have been linked to poor outcomes in treatment trials of depression and interventions targeting physical activity and/or sleep-wake cycle have been found to reduce depressive symptoms [18, 20, 21, 46, 47]. Moreover, an irregular sleep-wake cycle, as assessed by self-report and actigraphy measures, was reported in adolescents with a parental history of depression [48] and to prospectively predict depressive symptoms in adolescents [49].

As reviewed above, physical inactivity, sleep-wake, and circadian disturbances are increasingly acknowledged as crucial interrelated components of depression; however, it remains unclear whether they are associated with depression vulnerability. To address this issue, the present study aimed to estimate physical activity, sleep-wake, and circadian rhythms in young adults with and without a familiar risk for depression with the employment of wrist-worn actigraphy measured over a 7 days period. More specifically, sleep and physical activity patterns were examined as vulnerability markers. Considering that the levels of physical activity have been found to vary considerably between weekends and weekdays (i.e., greater physical activity during weekdays [50]), data collected during the week and weekend were treated separately. The formulated hypothesis was twofold and based on the existing literature. Particularly, the group with a familial risk for depression was expected to show (1) reduced estimates of physical activity (acceleration and MVPA) and (2) reduced sleep quality (efficiency) and reduced relative amplitude compared to the group without familial risk for the condition.

## Methods

### Participants

Based on previous papers on samples with clinical depression [25–27], a sensitivity power analysis in G*Power [51] for a repeated measures analysis of variance with one within-subject factor with two levels (weekdays vs. weekend days) and one between-subject factor with two levels (with a familial risk vs. without a familial risk) was performed to determine whether the sample size was large enough to detect a significant effect. This analysis revealed that the adequate total sample size to detect a moderate effect size (Cohen’s *f* = 0.22) with a power of 0.80 was 46 participants.

To identify young adults with and without familial risk for depression and no current depressive symptoms, a total of 163 university students from the University of Padua (Italy) participated in a first screening. College students were selected as participants for this study as they are expected to share most daily schedules, academic demands, and social environments, thus reducing potential confounding variables. The screening involved the completion of an online form that included the Beck Depression Inventory-II (BDI-II; [52, 53]) and ad-hoc questions regarding psychopathology in their first-degree relatives (e.g., “*has some of your first-degree relatives been diagnosed with or experienced depression*?”). Individuals who obtained a BDI-II score equal to or less than 12 and reported the presence of lifetime depression in at least a first-degree relative without reporting a history of other psychopathology and those who did not report any familiarity with psychopathology were invited to the Psychophysiology Laboratory of the Department of General Psychology of the University of Padua to conduct an experimental session (*n* = 71). Among the students that were invited to the experimental session, a total of 52 accepted and participated in this study.

The sample was medically healthy and free from psychotropic medication, as assessed with an ad-hoc anamnestic interview. The module A of the Structured Clinical Interview for DSM-5 (SCID 5-CV [54, 55]) was employed to assess the presence of current and past depressive symptoms, while the Family History Screen (FHS [56]) was administered to assess the presence of current or past depression and/or other psychopathological conditions in first-degree relatives. The FHS is a widely employed and reliable tool to assess the presence of a family history of depression and other psychopathological conditions (e.g., [57–59]). All the enrolled participants had a BDI-II score lower than 12 and did not meet the diagnostic criteria for a major depression episode, persistent depressive disorder, or bipolar disorder. However, individuals that experienced a past depressive episode (as assessed through the SCID-5-CV) were included in the group with a familial risk for depression as several of the young adults with a first-degree relative with a history of depression had already developed depressive symptoms throughout their lifetime (*n* = 8, none even treated pharmacologically)^1^

Based on the FHS, 20 participants with at least one first-degree relative with a history of depression were included in the group with a family history of depression (13 females; age = 22.7 ± 2.2), while 32 participants whose first-degree relatives did not have a family history of any psychiatric disorder comprised the group without a family history for depression (21 females; age = 22.0 ± 1.3). The two groups were matched in terms of sex, age, and years of education. Informed consent was obtained from all participants. The research was conducted in compliance with the World Medical Association Declaration of Helsinki on research on human subjects and was approved by the Ethical Committee of Psychological Research, Area 17, University of Padova (prot. no. 5007).

### Procedure

The current investigation is part of a larger study aimed at exploring depression vulnerability in young adults. For this investigation, at the end of an experimental session as a part of the larger study, participants were asked to wear a wrist actigraphy device for seven consecutive days and instructed to complete a daily sleep diary every morning. Specifically, the sleep diary consisted of participants responding to questions regarding to the timing and quality of their bed- and wake time and sleep-wake cycle as well as their diurnal activities associated with physical engagement (e.g., sports, going out, working). After 7 days, participants returned the actigraphy device and the diaries, were debriefed about the study, and given a graphical report of their sleep-wake cycle. Participants were also asked to complete two questionnaires investigating their self-reported sleep quality and chronotype.

### Psychological measures

The Italian version of the mood episode module (module A) of the SCID-5-CV was employed as a reliable tool to assess the presence of depression and to exclude individuals with depressive symptoms, persistent depressive disorder, or bipolar disorder. The SCID-5-CV was administered by a trained psychologist with experience administering structured clinical interviews. The Italian version of the BDI-II was also employed as a reliable measure of the severity of depressive symptoms in the past two weeks. It is a self-report questionnaire composed of 21 items, each with a Likert scale of four points and scores range from 0 to 63, where higher scores indicate greater depressive symptoms. In the Italian version, a score of 12 has been reported as the optimal cut-off score to discriminate between individuals with and without depressive symptoms.

The Italian translation of the FHS was used to assess the presence of family psychiatric conditions in first-degree relatives (i.e., biological parents, and siblings). An affirmative answer to item 8 (“*Did one of your parents or siblings ever have a period of feeling sad, blue, or depressed for most of the time for at least two weeks? Please answer by reporting the member of your family who experienced these feelings without including time of physical illness or mourning after a death*”) and/or to item 9 (“*Did one of your parents or siblings ever have a period (at least two weeks) of feeling quite tired, having less energy, or not caring about their usual activities? Please answer by reporting the member of your family who experienced these feelings without including time of physical illness or mourning after a death*”) was considered as indicative of a first-degree relative with a history of depression.

Circadian preferences were assessed using the Italian and reduced version of the Morningness–Eveningness Questionnaire (MEQr [60, 61]). The MEQr assesses self-reported chronotypes using five items, with scores ranging from 4 to 25, which categorize participants into evening (scores < 11), intermediate (scores between 11 and 18), and morning types (scores > 18).

The presence of insomnia was assessed with the Italian version of the Insomnia Severity Index (ISI [62]). The ISI comprises seven items targeting the subjective symptoms and daytime consequences of insomnia as well as the degree of distress caused by these difficulties. Scores range from 0 to 28, whereby higher scores indicate greater insomnia severity.

### Actigraphy recording and analyses

Participants’ motor activity was assessed for seven consecutive days using the AX3 triaxial Axivity accelerometer on their non-dominant wrist (Axivity, Axivity Ltd, Newcastle, UK). Participants were instructed to wear the watch day and night and the accelerometer was set to sample at 100 Hz. Data from participants wearing the watch for at least 16 h per 24 h time period were included in the analyses.

Raw actigraphy data were analysed with GGIR [63], an open-source R package. According to previously published methods [64], the processing steps included the verification of sensor calibration error using local gravity as a reference, detection of sustained abnormally high values, non-wear detection, and extraction of actigraphy-based physical activity and sleep measures.

Physical activity was estimated as daily gross motor activity (i.e., acceleration) and minutes in moderate-to-vigorous physical activity. Actigraphy-based gross motor activity was estimated by calculating the Euclidian Norm Minus One (ENMO: √(x^2 + y^2 + z^2) − 1g, 1 g = 9.81 m/s^2^), expressed in milligravity (mg), and averaged over 5 s epochs [65]. Specifically, the Euclidian Norm represents the overall magnitude of acceleration in three-dimensional space (x, y, and z axes). By subtracting one from this value, ENMO provides a measure of physical activity intensity while controlling for the influence of gravitational acceleration. Based on recent studies, minutes in moderate-to-vigorous physical activity per day were defined as the sum of 5 s epochs in which ENMO was larger than 100 mg (e.g., [65, 66]).

Sleep-wake cycle quality was assessed using an algorithm implemented in GGIR, considering that young adults tend to inconsistently comply with the completion of the daily sleep diary [67]. A Sleep Period Time-window was defined as the time window starting at sleep onset and ending when waking up after the last sleep episode of the night [67]. Inactivity periods overlapping the Sleep Period Time-window were labelled as sleep periods. Moreover, total sleep duration (in hours) was calculated as the sum of estimated sleep periods. Finally, sleep efficiency [%] was computed as follows:

$${Sleep\; efficiency}( \% )={Total\; Sleep\; Time}/{Total\; Time\; in\; Bed}\times 100$$

Where Total Sleep Time is the duration of actual sleep during the recording period and Total Time in Bed is the total duration spent in bed, including both sleep and wake periods (in hours). This metric is expressed as a percentage, with higher values indicating a more efficient use of time in bed for sleep [67].

Circadian rhythms were assessed by the computation of relative amplitude (RA), a measure that reflects the extent of variation between the peak activity periods (usually during wakefulness) and the troughs (typically during sleep) within the 24 h cycle. To compute RA, the most active 10 h of the day (M10) are compared with the least active 5 h of the night (L5; i.e., M10 minus L5 divided by M10 plus L5). Particularly, lower relative amplitude relates to a dampened circadian rhythms amplitude and suggests lower activity during the day and greater activity during the night. Relative amplitude ranges from 0 to 1, where higher values indicate a rhythm with higher amplitude. Moreover, the inter-daily stability was computed (i.e., the stability of the 24 h activity rhythm across the seven-day time window), whereby scores can range from 0 to 1, with higher scores indicating more stable rhythms e.g., [68].

### Statistical analyses

Statistical analyses were performed with Jamovi (The Jamovi Project, 2021). Distributions of all variables were checked on normality with QQ plots. Demographics and psychological variables were compared between the group with a familial risk for depression and the group without familial risk for depression using independent-sample *t-*tests and *χ*2. A *p*-value < 0.05 was considered statistically significant.

For each physical activity and sleep measure of interest (i.e., acceleration, MVPA, total sleep duration, sleep within sleep period time, and sleep efficiency, relative amplitude), a 2 × 2 ANOVA with Group (with familiarity vs. without familiarity) as a between-subjects factor and Day Type (Weekdays: Monday thru Friday; Weekends: Saturday and Sunday) as a within-subjects factor [69]. The Tukey HSD test was used for post-hoc comparisons, and partial eta squared (ηp2) was reported as an estimate of effect size. Then, correlations between actigraphy-derived sleep estimates and self-reported sleep quality (ISI) were conducted using Pearson correlations.

## Results

### Demographics and psychological measures

Demographic and psychological measures are reported in Table 1. The two groups did not differ in terms of BMI, BDI-II, and ISI scores. The sample was characterized by a high prevalence of intermediate circadian preferences (*n* = 37, 71.2%), with five (9.6%) participants defined as evening types and the remaining ten (19.2%) as morning types. No significant differences between the two groups could be detected in circadian preferences, alcohol consumption, or smoking habits. Finally, in line with previous investigations (e.g., [67]), participants were found to inconsistently comply with the completion of precise the daily sleep diary, whereby the adherence to the precise timing of sleep indications was not consistent.

**Table 1:** Demographic and psychological variables for the two groups.

	Group without a familial risk (*n* = 32)	Group with a familial risk (*n* = 20)	*p*
Age	22.0 (1.3)	22.7 (2.2)	.17
Female Sex (%)	65.6	65.0	.97
Education (years)	15.0 (1.6)	15.0 (1.5)	.95
BMI	22.4 (2.3)	21.7 (3.9)	.54
BDI-II	3.5 (2.9)	4.7 (3.0)	.18
ISI	4.8 (3.9)	5.2 (3.8)	.72

Data is shown in mean (standard deviation).

*BMI* body mass index, *BDI-II* Beck depression inventory, *ISI* insomnia severity index.

### Actigraphy estimates of physical activity

**Fig. 1:**
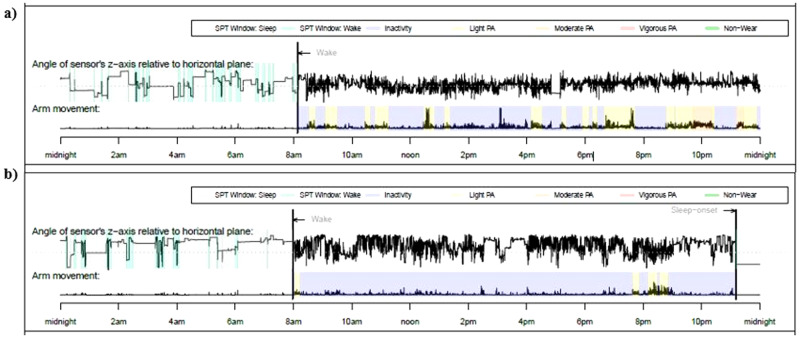
Raw actigraphy data. 24 h data of one participant without (**a**) and one participant with a familial risk for depression (**b**). The graphs show acceleration in the x, y, and z axis from which physical activity and sleep patterns were identified. Activity counts per minute are represented by the height of the black lines. Colors represent the levels of physical activity as identified by GGIR algorithms with specific acceleration cut-points (sedentary time (purple, <30 mg), light (yellow, 30–100 mg), moderate (orange, 100–400 mg), and vigorous (red, ≥400 mg) physical activity). In line with the results, the participant with a familial risk for depression presents with reduced physical activity and predominant inactivity relative to the participant without a familial risk for depression.

**Fig. 2:**
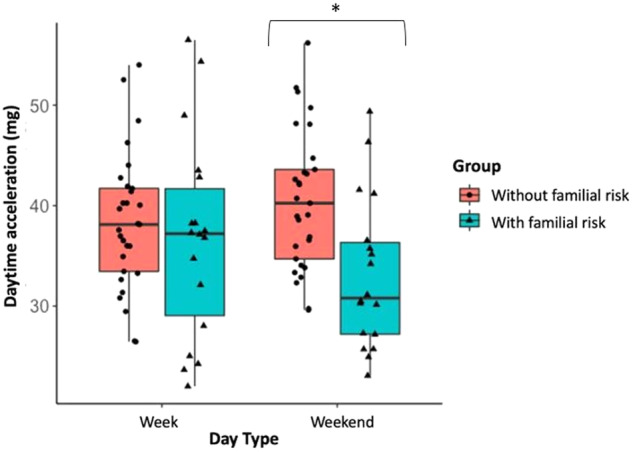
Daytime acceleration boxplot. Boxplot of the acceleration (mg) for week and weekend days in the group with a familial risk for depression (in light blue) and the group without a familial risk for depression (in pink). *p ≤ .05.

**Fig. 3:**
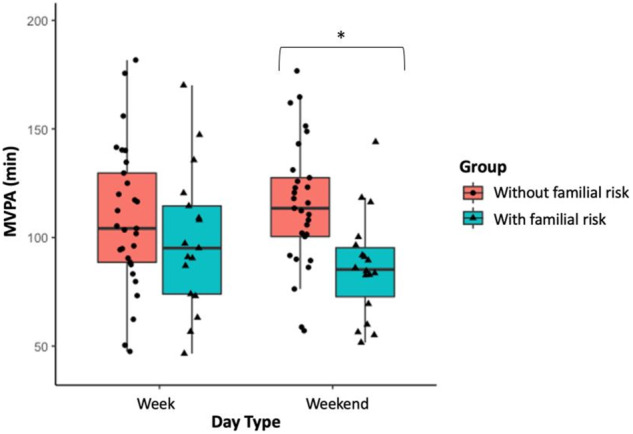
MVPA boxplot. Boxplot of the time spent doing moderate-to-vigorous physical activity (MVPA, in minutes) for week and weekend days in the group with a familial risk for depression (in light blue) and the group without a familial risk for depression (in pink). *p ≤ .05.

Due to battery or technical problems, data from three participants (two controls, one with a familial risk for depression) were not available for analysis. Actigraphy-based estimates are reported in Table 2 for the two groups separately. The assumption of homogeneity of variances across groups was upheld through a Levene’s test for every employed ANOVA (all *P*s > .15). The sample had moderate inter-daily stability (mean = 0.6 ± 0.1), with no statistical difference between the two groups (*t*_(46)_ = −0.62, *p* = .54). For explicatory purposes, Fig. 1 provides a visual inspection of the output of 24 h actigraphy data for two individuals from our study (one from each group). The ANOVA on acceleration yielded a Group effect (F_1,45_ = 5.15, *p* = .03, ${\eta }_{p}^{2}$ = 0.10), where the group with a familial risk for depression showed reduced gross motor activity compared to the group without a familial risk. Moreover, as shown in Fig. 2, a significant Day Type × Group effect emerged (F_1,45_ = 6.03, *p* = .018, ${\eta }_{p}^{2}$ = 0.12), where the group with a familial risk for depression showed reduced gross motor activity compared to the group without a familial risk for depression only during weekends (*p* = .01), whereas no difference emerged on weekdays. that emerged for time spent engaged in MVPA (F_1,45_ = 6.17, *p* = .02, ${\eta }_{p}^{2}$ = 0.12), such that the group with a familial risk for depression spent less time engaging in MVPA compared to the group without a familial risk for depression during weekends (*p* = .006), whereas there were no significant differences that could be detected on weekdays (Fig. 3)^2^.

**Table 2:** Actigraphy-based estimates of physical and sleep activity in the two groups.

	Group without a familial risk (*n* = 30)	Group with a familial risk (*n* = 19)	*p*
Week Days			
Daily acceleration (mg)	38.8 (7.2)	36.7 (10.1)	.41
MVPA (minutes)	112 (36)	105 (42)	.60
Total sleep duration (hours)	7.6 (0.7)	8.0 (0.6)	**.04**
Sleep within sleep period time (hours)	6.8 (0.6)	7.1 (0.6)	.07
Sleep efficiency (%)	91.0 (0.4)	89.0 (0.5)	.20
Relative amplitude	0.8 (0.1)	0.8 (0.1)	.43
Weekend Days			
Daily acceleration (mg)	40.5 (6.9)	33.1 (7.6)	**<.001**
MVPA (minutes)	115 (29.2)	86.6 (23.9)	**<.001**
Total sleep duration (hours)	7.7 (1.2)	7.8 (1.0)	.70
Sleep within sleep period time (hours)	6.9 (0.9)	7.1 (1.1)	.53
Sleep efficiency (%)	90.0 (0.4)	90.0 (0.4)	.53
Relative amplitude	0.9 (0.1)	0.9 (0.1)	.73

Data is shown in mean (standard deviation).

*MVPA* moderate-to-vigorous physical activity. Significant effects are shown in bold.

### Actigraphy estimates of the sleep-wake cycle and circadian rhythms

Actigraphy-based estimates of the sleep-wake cycle are reported in Table 2 for the two groups separately. Regarding sleep-wake cycle and circadian rhythms, participants tended to go to bed on average at 00:50 ± 1:19 and wake up at 08:36 ± 1:09. The sample spent on average 7 h and 45 min ± 42.6 min in bed with 87.5% of the sample showing a Time in Bed (TIB) longer than 7 h. No significant difference between the two groups were detected for any of these sleep parameters. The ANOVA on sleep efficiency showed a Day Type × Group effect (F_1,45_ = 7.04, *p* = .01, ${\eta }_{p}^{2}$ = 0.14); however, Tukey *post-hoc* tests did not show any significant comparison. In terms of circadian rhythms, no Day Type, Group or Day Type × Group effect emerged in terms of relative amplitude. Finally, actigraphy-based sleep estimates and self-reported sleep quality (ISI) were not significantly correlated.

## Discussion

The present study aimed to explore whether physical activity, sleep-wake cycle, and circadian rhythms alterations might represent an early indicator of depression vulnerability in a group with a familial risk for depression but no current depression relative to a control group (without familial risk). Actigraphy estimates were gathered over seven days in a sample of young adults with and without familial risk for depression. As expected, the at-risk group showed reduced physical activity estimates. By contrast, the at-risk group did not differ in terms of sleep-wake cycle and circadian rhythms estimates compared to the control group.

In line with findings from clinical samples (e.g., [25, 31]), young adults with a first-degree relative with a history of depression showed reduced physical activity, as indicated by reduced daily acceleration estimates and time spent engaged in moderate-to-vigorous physical activity. This pattern was marked for weekends, suggesting that non-clinical vulnerable individuals might particularly withdraw from engaging in activities during their free time away from scheduled activities. Overall, these results indicate that a lower level of daily motor activity is not only a feature of mood disorders [28, 70] but might be a manifestation of early risk. These results support the involvement of the Sensorimotor Systems of the RDoC in depression risk, suggesting that actigraphy-based assessment could be a useful and feasible tool to integrate into clinical assessment aimed at identifying those that are at a higher risk for the disorder. Considering the tight link between physical activity and the likelihood of engaging in behaviors associated with rewarding and pleasant outcomes (e.g., meeting friends or experiencing an amusing event), the interrelation between the Sensorimotor Systems and the Positive Valence Systems becomes evident. As a matter of fact, positive emotionality and approach motivation, dimensions that appear to be blunted in individuals at risk for depression (e.g., [4, 59, 71, 72]), support action engagement (mostly during leisure time) and might be concurrently involved in determining depression risk. In addition, reduced physical activity may be a mechanism linking not only depression risk to approach motivation deficits but also to negative health outcomes and chronic diseases that are often comorbid with depression (i.e., cardiovascular diseases, diabetes) [73, 74]. Overall, reduced diurnal rhythms, especially on unstructured days, might represent a vulnerability indicator of depression even before the development of depression or potential relapse into an episode.

Given the tight link between daytime activity and the sleep-wake cycle, actigraphy-based sleep and circadian alterations were also expected to be involved in the at-risk sample of young adults. The hypothesis was based on several studies showing reduced sleep quality in clinical samples (e.g., [26, 34, 41, 48]), assessed mostly with self-reported measures but also with wrist-worn actigraphy. However, contrary to the hypothesis, those with a familial risk for depression did not show actigraphy-based or self-reported sleep-wake or circadian disturbances. Indeed, sleep disturbances might become evident at subsequent stages of depression development, without necessarily having to be a prodromal sign or risk of the condition. In line with this, Difrancesco and colleagues (2019) found that only individuals with severe, but not mild, depressive symptoms showed actigraphy-based reductions in sleep quality (i.e., sleep duration and efficiency). Considering the variability and polarization of sleep symptoms (e.g., insomnia and hypersomnia) in depression, it may be difficult to explore this dimension, especially in depression risk.

From a clinical perspective, the present findings offer novel insights into viable and ecologic indicators of risk that could serve as modifiable targets to prevent full-blown depression. For instance, physical inactivity, which was a feature of familial risk for depression, represents a modifiable factor that might be targeted and offered as an adjunctive treatment to the already employed cognitive-behavioral early interventions. For example, a useful evidence-based treatment for depression [75] and at-risk populations [76] is behavioral activation therapy, a strategy to increase engagement in enjoyable activities and positive interactions between the individual and the environment [75, 77]. Embedding structured physical activity and exercise training into behavioral activation and using actigraphy to monitor progress could provide a feasible and ecological way to prevent depression onset while minimizing in-person visits, thus increasing participation (e.g., [78]). Indeed, literature shows that even small increases in physical activity reduced the likelihood of future depressive episodes in cohorts of remitted individuals [19]. Similarly, short exercise-based training improved depressive symptoms and approach motivation, as assessed by self-report measures [18–22] and neural responses to appetitive images [79, 80]. On the contrary, the results of this study suggest that sleep interventions may be specifically effective in individuals with already-developed depressive symptoms [26].

In interpreting the results of this study, some limitations should be considered. Firstly, data on class attendance, lecture start time, and academic performance could have been useful to consider but were not collected. Moreover, the sample size was relatively small, limiting more specific analyses or additional covariates inclusion. Lastly, the cross-sectional nature of the study did not allow to determine the extent to which physical activity is linked to depression onset, and longitudinal investigations will have to be conducted.

Taken together, this was the first study to examine actigraphy-based physical activity, sleep-wake, and circadian disturbances in young adults with a familial risk for depression. What emerged was a pattern of reduced physical activity (daily acceleration and time spent doing moderate-to-vigorous activity), especially during unstructured days (i.e., weekends), but no circadian disturbances, in those with a higher risk of developing depression. To our knowledge, this study was the first to adopt an RDoC approach to study the sleep-wake cycle with wrist-worn actigraphy in depression vulnerability and results highlight the importance of motor behavior and the Sensorimotor domain of the RDoC in vulnerability to this condition. Future studies should consider integrating actigraphy-based estimates of physical activity into early identification and prevention strategies.

## Data Availability

Data and R code used to obtain actigraphy-based measures through the GGIR package will be made available upon request.

## Footnotes

1 Within the group with a familial risk for depression, participants that previously had a past depressive episode did not differ from those that did not have a past depressive episode in all actigraphially-derived measures (all *Ps* > .07).

2 It is noteworthy that one participant belonging to the group with familiar risk engaged in night-time employment during the recording week (particularly in the weekend), whereas the other participants did not report working during that time window. While this could not be accounted for as a covariate in the analyses, an additional analysis was conducted by excluding this participant from the dataset. Importantly, this removal did not yield any substantive changes to the main statistical results.
